# RNA-Seq and 16S rRNA Analysis Revealed the Effect of Deltamethrin on Channel Catfish in the Early Stage of Acute Exposure

**DOI:** 10.3389/fimmu.2022.916100

**Published:** 2022-06-03

**Authors:** Yibin Yang, Xia Zhu, Ying Huang, Hongyu Zhang, Yongtao Liu, Ning Xu, Guihong Fu, Xiaohui Ai

**Affiliations:** ^1^ Yangtze River Fisheries Research Institute, Chinese Academy of Fishery Sciences, Wuhan, China; ^2^ Fishery Resource and Environment Research Center, Chinese Academy of Fishery Sciences, Beijing, China; ^3^ College of Animal Science and Technology, Hunan Agricultural University, Changsha, China

**Keywords:** channel catfish, deltamethrin, immunotoxicity, metabolic disorder, apoptosis

## Abstract

Deltamethrin (Del) is a widely used pyrethroid insecticide and a dangerous material that has brought serious problems to the healthy breeding of aquatic animals. However, the toxicological mechanisms of Del on channel catfish remain unclear. In the present study, we exposed channel catfish to 0, 0.5, and 5 μg/L Del for 6 h, and analyzed the changes in histopathology, trunk kidney transcriptome, and intestinal microbiota composition. The pathological analyses showed that a high concentration of Del damaged the intestine and trunk kidney of channel catfish in the early stage. The transcriptome analysis detected 32 and 1837 differentially expressed genes (DEGs) in channel catfish trunk kidneys after exposure to 0.5 and 5 μg/L Del, respectively. Moreover, the KEGG pathway and GO enrichment analyses showed that the apoptosis signaling pathway was significantly enriched, and apoptosis-related DEGs, including cathepsin L, p53, Bax, and caspase-3, were also detected. These results suggested that apoptosis occurs in the trunk kidney of channel catfish in the early stage of acute exposure to Del. We also detected some DEGs and signaling pathways related to immunity and drug metabolism, indicating that early exposure to Del can lead to immunotoxicity and metabolic disorder of channel catfish, which increases the risk of pathogenic infections and energy metabolism disorders. Additionally, 16S rRNA gene sequencing showed that the composition of the intestinal microbiome significantly changed in channel catfish treated with Del. At the phylum level, the abundance of Firmicutes, Fusobacteria, and Actinobacteria significantly decreased in the early stage of Del exposure. At the genus level, the abundance of *Romboutsia, Lactobacillus*, and *Cetobacterium* decreased after Del exposure. Overall, early exposure to Del can lead to tissue damage, metabolic disorder, immunotoxicity, and apoptosis in channel catfish, and affect the composition of its intestinal microbiota. Herein, we clarified the toxic effects of Del on channel catfish in the early stage of exposure and explored why fish under Del stress are more vulnerable to microbial infections and slow growth.

## Introduction

Pyrethroids are synthetic pesticides that were first isolated from the flower extract of *Chrysanthemum*. Due to its selectivity and low toxicity to non-target organisms, such as mammals and birds, pyrethroids are widely used to control agricultural and residential pests (1–4). However, pyrethroids are highly toxic to aquatic animals since they lack the corresponding degrading enzymes (5). Additionally, due to the lipophilicity of pyrethroids (6, 7), in the aquatic environment, they can easily enter into fish’s bodies through gills and are transported to each tissue through blood circulation and accumulate. Previous studies have shown that the LD_50_ of pyrethroids to aquatic animals is 10 to 1000 times lower compared to mammals and birds (8–11). Therefore, the pollution of pyrethroids can harm aquatic animals. Deltamethrin (Del) is an important pyrethroid with a broad-spectrum insecticidal activity that is widely used in aquatic animal pest control (12). Del can flow into rivers and lakes through surface runoff and domestic wastewater, threatening the health of aquatic organisms, including fish (13, 14). For example, Feoet al. reported that the concentration of Del in water samples from the Ebro River Delta in Spain was between 0.73 - 58.80 ng/L, and Amin et al. indicated a concentration in water samples of 0.20-2.00 μg/mL (15, 16). There have also been reports of a large number of deaths of aquatic animals caused by Del pollution (17). Hence, considering the high toxicity and environmental residues of Del, its harmful effects on aquatic organisms have attracted increasing attention.

Furthermore, Del has been proved to be highly toxic to different aquatic animals, including Chinese mitten crab *(Eriocheir sinensis*), Chinese rare minnow (*Gobiocypris rarus*), black tiger shrimp (*Penaeus monodon*), zebrafish (*Danio rerio*), and Tilapia mossambica (*Oreochromis niloticus*) (12, 18–24). Meanwhile, Del is an important stress source for freshwater ecosystems (20, 25). The potential health risks and toxicity of Del are mainly related to neurotoxicity and hepatorenal toxicity (26, 27). According to previous studies, Del can inhibit the activities of acid phosphatase, lysozyme, and phenoloxidase in crayfish (26). Long-term exposure to Del might also induce inflammation, oxidative stress, and apoptosis in different organs of carp in a dose-dependent manner (28). Del might also lead to developmental toxicity, neurotoxicity, and cardiovascular toxicity in zebrafish (29, 30). Additionally, subacute exposure to environmentally relevant concentrations of Del can inhibit the host immune response and disease resistance of snakehead fish (*Channa argus*) (24). Besides, Del exposure leads to histopathological changes and immunotoxicity of Chinese rare minnows, making them vulnerable to pathogen infections (31), and can cause gill tissue damage and immunotoxicity in crucian carp (12). These results demonstrated that different degrees of exposure to Del can have toxic effects on aquatic animals, especially on the immune system. Therefore, Del is a dangerous material, but its harmful molecular mechanisms remain unclear.

In the past decade, RNA-seq has been widely used in aquatic toxicology research to explore potential functional gene networks and gene mining (32). RNA-seq has also been used as a visualization tool to reveal the whole gene expression of immune response and signaling pathways of fish in specific physiological processes. Thus, we can explore the transcriptional landscape at the overall level and better describe the potential molecular mechanisms of Del-induced toxicity (33). The trunk kidney is an important organ for fish to transform and excrete exogenous substances. Meanwhile, it also plays a key role in immune response and maintaining homeostasis (34, 35). Hence, acute exposure to Del will inevitably cause a strong trunk kidney response. Transcriptome changes in the trunk kidney are of great significance to reveal the toxic mechanisms of Del in fish. The intestinal microbiota is also crucial to fish health and plays a key role in maintaining host physiology, nutrient supply, and intestinal immune response (36). Previous studies have shown that various environmental pollutants can cause changes in the composition of the intestinal microbial community, thereby affecting the health of aquatic animals (37, 38). Therefore, under acute exposure to Del, the composition of fish intestinal microbiota will change to adapt to the new environment and have an important impact on the host life activity processes. Thus, it is necessary to clarify the changes in fish intestinal microbiota under Del exposure to better understand its toxic effects.

Channel catfish (*Ictalurus punctatus*) was introduced in China from the United States in 1984. Due to their adaptation to the breeding environment in China, the breeding output and area have explosive growth, with an annual output of 308000 tons, becoming one of the important varieties of freshwater fish cultured with Chinese characteristics. However, diseases have always been the bottleneck restricting the further development of the channel catfish industry. Pathogenic microorganisms that infect channel catfish, including *Aeromonas hydrophila*, *Yersinia ruckeri*, *Edwardsiella ictalurid*, and *Streptococcus iniae*, have caused great economic losses to the channel catfish breeding industry (39, 40). Additionally, the inhibition of the immune system of channel catfish is an important cause of pathogenic microbial infections. Therefore, in the present study, we aimed to explore the toxic mechanisms of Del on channel catfish by analyzing the changes in trunk kidney transcriptome and the intestinal microbiome. We focused on the toxic effects of Del on channel catfish immune system to provide the theoretical basis for disease prevention and control.

## Materials and Methods

### Reagents and Fish

Deltamethrin (purity > 98%) was obtained from Sigma Aldrich (Chemical Co., USA), then dissolved in dimethyl sulfoxide (DMSO) as a stock solution. All healthy juvenile channel catfish (100 ± 20 g) were purchased in the Wuhan Baishazhou aquatic market. Before the experiment, these fish were domesticated for one week under laboratory conditions free of specific pathogens at 25 ± 1°C.

### Experimental Design and Samples Collection

Ninety healthy channel catfish were randomly selected and divided into three groups, with three replicates in each group and 10 fish in each replicate. According to the pre-test results about lethal concentration of deltamethrin on channel catfish and the residual amount of Del in the nature water environment, 0.5 (L group) and (H group) 5 μg/L of Del were selected to study its toxic effect on channel catfish. The control group (C group) was soaked with DMSO. The final concentration of DMSO in experimental and control groups was under 0.001% (v/v). Experimental fish were not fed throughout the exposure period. After 6 h of exposure, 15 channel catfish were selected from each group to collect intestinal contents. Five intestinal contents were mixed to form a sample. The intestinal contents were squeezed into sterile centrifuge tubes, immediately frozen in liquid nitrogen, then stored at - 80°C for DNA extraction. Meanwhile, trunk kidneys were taken from 15 randomly selected channel catfish in each group, and five trunk kidneys were mixed to form a sample. Then, samples were immediately frozen in liquid nitrogen and stored at - 80°C for further transcriptomics. Additionally, the trunk kidneys and intestines of channel catfish in each group were cut and put into 10% neutral formaldehyde to make paraffin sections and stained with Hematoxylin and Eosin (HE) to study the pathological damage of Del to the intestines and trunk kidneys of channel catfish.

### RNA Extraction, Library Construction, and Sequencing

Nine trunk kidney samples (three samples per treatment) were selected for RNA sequencing (RNA-seq). After total RNA was extracted, enriched mRNA by Oligo(dT) beads were fragmented using fragmentation buffer and reverse transcripted into cDNA with random primers using the NEBNext^®^ Ultra™RNA library prep kit for Illumina following the manufacturer’s instructions. For the first-strand cDNA synthesis, the fragmented and primed mRNA was reversed to cDNA in a 20 μL reaction using protoscript II reverse transcriptase at 25°C for 10 min, then at 42°C for 15 min, and at 70°C for 15 min. For the synthesis of the second strand cDNA, the second strand synthase mixture was added to the first strand synthesis reaction at 16°C for 1 h with 80 μL. Then, the cDNA fragment was purified using the QiaQuick PCR Extraction Kit (Qiagen, Germany), and the end repair and poly (a) were performed. Finally, the Illumina sequencing adapter was connected. The ligation products were selected by size using agarose gel electrophoresis, PCR-amplified, and sequenced using Illumina HiSeq2500 by Genedenovo Biotechnology Co, Ltd (Guangzhou, China). For RNA-seq of fish samples under sterile conditions, libraries were sequenced on the Illumina HiSeq Xten platform.

### Bioinformatic Analyses

Adaptor sequences were trimmed by cutadapt with at least 30 nt of remaining length. Clean reads were mapped to the channel catfish reference genome using HISAT2 (41). Gene expression levels were estimated by the number of fragments per million fragments per thousand base transcripts (FPKM). DESeq2 was used for differential expression analysis. The threshold of differentially expressed genes (DEGs) was set as FDR < 0.05 and |log_2_ [fold change (FC)]| > 1 (42). The gene ontology (GO) enrichment analysis of DEGs was performed with the “GOseq” R package, while the Kyoto Encyclopedia of Genes and Genomes (KEGG) pathway enrichment analysis was performed with KOBAS software (43).

### Quantitative Reverse Transcription-Polymerase Chain Reaction (qRT-PCR)

We used quantitative reverse transcription (qRT)-PCR to verify the expression level of DEGs identified by RNA-Seq. The primers used are shown in **Table 1** (39, 44–50). Channel catfish EF-1 α was used as the internal parameter to homogenize the expression levels. The qRT-PCR was performed using quantum Studio 6 flex (life technologies, USA), and melting curves were generated at the end of the run to confirm the specificity. The 2^−ΔΔCt^ method was used to calculate the relative levels of DEGs (51).

**Table 1:** Primers used in qRT-PCR for DEGs validation.

Primers	Sequence (5′ to 3′)	Application
TLR-1-F	AGCCAAAGAAATGCCAACTG	Real-time PCR
TLR-1-R	TGAAGTCTCGTTCGTGGTGA	Real-time PCR
pglyrp6-qF	GAGATGGATGAAGGGCTGAA	Real-time PCR
pglyrp6-qR	CACTGCTGGAAGGTCAGACA	Real-time PCR
egln3-qF	CCTCGGTGAAGCAATTGGTC	Real-time PCR
egln3-qR	ATGGCTTCGGATCCTCTCTC	Real-time PCR
VMO1-qF	AGCCCACAAATCCCAGTTCCA	Real-time PCR
VMO1-qR	GATGACACCGCTGCTAACAACA	Real-time PCR
iNOS-qF	TCAGCAGATGTCCGATGTCA	Real-time PCR
iNOS-qR	AGGAGTTCATTGGTGGAAGGT	Real-time PCR
Star-qF	AAGGTCGGAGATCAGATG	Real-time PCR
Star-qR	ATGAGTAGCAGAGTATGGT	Real-time PCR
g6pca.1-qF	TGTGGAGTTATCTTAGGTATCATT	Real-time PCR
g6pca.1-qR	CTTCAGGCTGGTGTTGTA	Real-time PCR
IL-1β-qF	GTGTAAGCAGCAATCCAGTCA	Real-time PCR
IL-1β-qR	CAAGCACAGAACAGTCAGGTAT	Real-time PCR
Hsp70-qF	CTTGATGTTACCCCTCTGTCTCT	Real-time PCR
Hsp70-qR	TCAGAGTAGGTGGTGAAAGTCTG	Real-time PCR
NOD1-qF	CCTTACACCCTGACCCCACC	Real-time PCR
NOD1-qR	CTTTTTCCCCCCTCTCTCTTTC	Real-time PCR
Nlrc3-qF	TGGCTTCCAAAACCACTATCG	Real-time PCR
Nlrc3-qR	ACCGCCTCGCCTCCTGAT	Real-time PCR
myosin-1-qF	TGATGACCCACCTCAGTGAA	Real-time PCR
myosin-1-qR	CACAGTGACGCAGAACAACC	Real-time PCR
PCNA-qF	ACCTCAGCAGTATGTCCAAG	Real-time PCR
PCNA-qR	CAGAGAGTCTGCATTGTCCT	Real-time PCR
mstna-qF	CTCGGGGACGACGGCAAG	Real-time PCR
mstna-qR	CTTGAACGTCGGGGTTGG	Real-time PCR
TLR21-qF	TTCCTCTGCAGTGAGTGGTG	Real-time PCR
TLR21-qR	TGTGTCCAGAACAGCTCCTG	Real-time PCR
TLR5-qF	TTGGAAGCGCTACAAATCCT	Real-time PCR
TLR5-qR	ACCCGGAGGTTGAATAATCC	Real-time PCR
TLR18-qF	GCGTGGTTAAGAGCGAAAAG	Real-time PCR
TLR18-qR	GGAAGGAAGTCTCGCTTGTG	Real-time PCR
C3-qF	AGTTGAATACCGCTGCCAAC	Real-time PCR
C3-qR	CTCTCCATGCGCTGAGTACA	Real-time PCR
EF-1α-qF	GTTGAAATGGTTCCTGGCAA	Real-time PCR
EF-1α-qR	TCAACACTCTTGATGACACCAAC	Real-time PCR

### DNA Extraction, PCR Amplification, and Sequencing

Bacterial DNA was purified using the TIANamp Bacteria DNA kit (Tiangen Biotech Inc., Beijing, China), following the manufacturer’s protocols. A region encompassing the V3–V4 hypervariable regions of the 16S rRNA gene was amplified using the primers 341F (CCTACGGGNGGCWGCAG) and 806R (GGACTACHVGGGTATCTAAT) (52). The PCR amplification system comprehended 20 μL, including 10 μL premixed Taq (Takara), 1 μL of each primer (10 μM), 2 μL gDNA, and 6 μL ddH_2_O. The PCR reaction procedure comprehended a pre-incubation at 95°C for 3 min, 30 cycles of 95°C for 30 s, 50°C for 30 s, 72°C for 1 min, and finally 72°C for 7 min. The amplified PCR products were separated by agarose gel electrophoresis and recovered by gel cutting and quantified by QuantiFluorTM fluorimetry. Purified amplification products were mixed in equal quantities, the sequenced connector was joined, the sequencing library was constructed, and Illumina PE250 was used for sequencing.

### Biodiversity Analysis

For microbiome data analysis, FASTP (https://github.com/OpenGene/fastp) was used to filter the original data containing adapters or low-quality readings, and paired-end clean reads were merged as raw tags using FLSAH (53), with a minimum overlap of 10 bp and mismatch error rates of 2%. Clean labels were searched according to the reference database, and the reference-based chimerism check was performed using the uchime algorithm (http://www.drive5.com/usearch/manual/uchime_algo.html). All chimeric labels were removed to finally obtain valid labels for further analysis. Effective tags were clustered into operational taxonomic units (OTUs) of ≥ 97% similarity using UPARSE (54). The tag sequence with the highest abundance was selected as the representative sequence within each cluster. A between-groups Venn analysis was performed using R (version 3.4.1) to identify unique and common OTUs. Representative sequences were classified into organisms through a naive Bayesian model using RDP based on the Silva database (https://www.arb-silva.de/). Chao1, Simpson, and other alpha diversity indices were calculated in QIIME. The OTU sparse curve and rank abundance curve were also drawn in QIIME. The microbial communities of different samples were evaluated by principal coordinate analysis (PCoA) and arithmetic mean group method (UPGMA) β Diversity analysis. Calculation and plot multivariate statistics were performed in R. The KEGG pathway analysis of OTU was inferred by picrust (55) and tax4fun (56).The microbiome phenotypes were classified using BugBase.

### Statistical Analyses

Data are expressed as means ± standard deviations. SPSS 22 (American SPSS company) was used for data analyses. Independent sample t-test, one-way analysis of variance (ANOVA), and Dunnett test were used to detect differences between groups. A *p* < 0.05 was considered statistically significant.

## Results

### Pathological Changes of Channel Catfish in the Early Stage of Del Exposure

**Figure 1:**
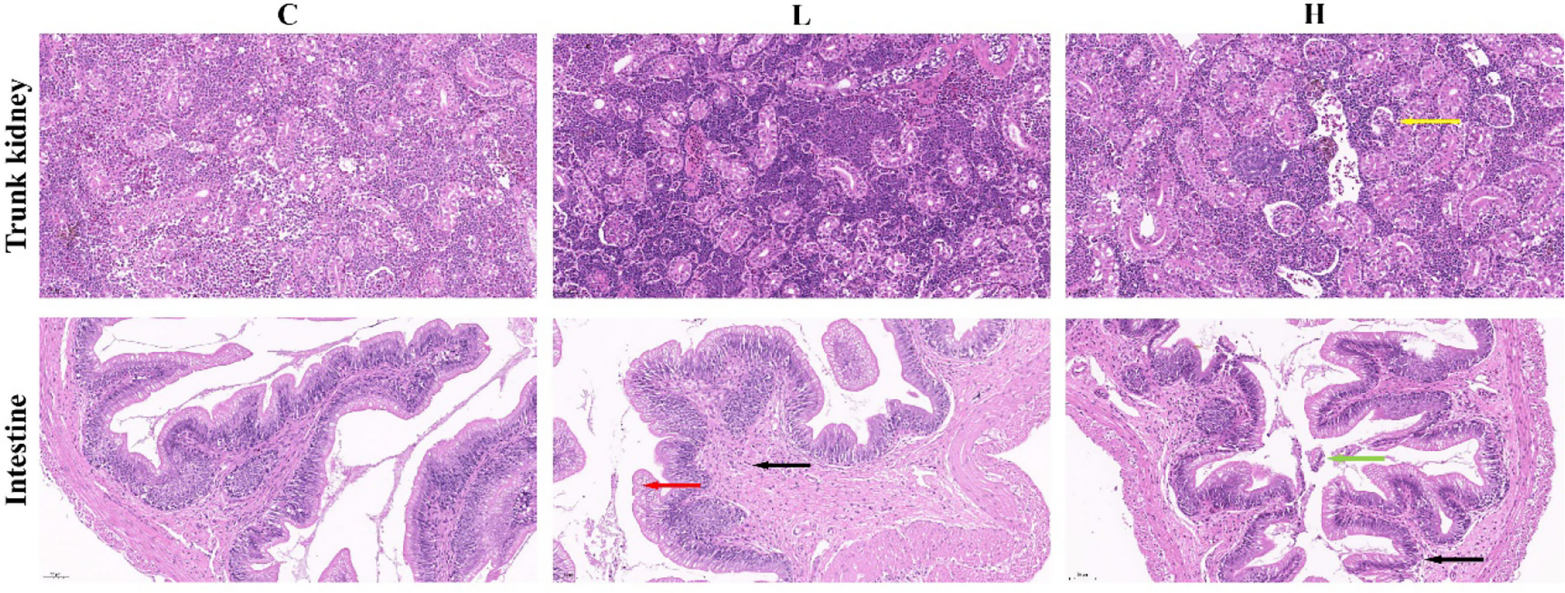
Pathological changes of the intestine and trunk kidney in different groups. Black arrows represent inflammatory cell infiltration; Red arrows represent edema and degeneration of intestinal chorionic epithelial cells; Green arrows represent the shedding of intestinal chorionic epithelial cells; Yellow arrows represent tubular atrophy and denaturation. C: 0 μg/L Del; L: 0.5 μg/L Del; H: 5 μg/L Del.

In the present study, we found that the trunk kidney and intestine of channel catfish in the H group presented different degrees of pathological changes, including a disordered arrangement of intestinal structure, disordered shedding of some intestinal villous epithelial cells, infiltration of inflammatory cells, and atrophy and degeneration of trunk kidney glomerulus. However, the trunk kidney glomerulus in controls (C group) and the L group was not significantly denatured, and the tissue was normal. Meanwhile, the intestine of L group fish showed edema and degeneration of some villous epithelial cells and infiltration of tissue inflammatory cells. On the other hand, the intestine of the C group was normal (**Figure 1**). The clinical symptoms of channel catfish in the C and L groups had no significant change in the activity state. Meanwhile, fish in the H group were belly up, unable to swim, floating, and spot bleeding on the body surface after Del exposure for 6 h.

### Transcriptomes

Nine cDNA libraries were constructed from channel catfish trunk kidneys and sequenced. The characteristics of these libraries are summarized in **Table 2**. After quality control of sequencing data, the number of clean reads of each library ranged from 40330662 to 56410298, and the Q30 value of each sequencing library was higher than 93.41%. Then, clean reads were mapped to the reference genome (http://ftp.ensembl.org/pub/release-105/fasta/ictalurus_punctatus/) and presented mapping rates ranging from 88.70 to 89.92% in different libraries.

**Table 2:** Summary statistics of transcriptome sequences.

Sample	Clean reads	GC content (%)	Q30 (%)	Uniquely mapped reads (ratio)	Multiple mapped reads (ratio)	Total Mapped reads (ratio)
**C-1**	44565422 (99.71%)	3170199478 (47.59%)	6242183498 (93.71%)	38123141 (85.64%)	1586743 (3.56%)	39709884 (89.21%)
**C-2**	44524246 (99.67%)	3141435335 (47.18%)	6232393709 (93.59%)	37925730 (85.30%)	1513009 (3.40%)	39438739 (88.70%)
**C-3**	43833092 (99.66%)	3081414867 (47.00%)	6124384817 (93.41%)	37897050 (86.52%)	1489102 (3.40%)	39386152 (89.92%)
**L-1**	41231736 (99.62%)	2921445041 (47.39%)	5765766272 (93.53%)	35519284 (86.20%)	1304274 (3.17%)	36823558 (89.36%)
**L-2**	56410298 (99.68%)	3998116385 (47.42%)	7906777742 (93.78%)	48465310 (86.02%)	1900187 (3.37%)	50365497 (89.39%)
**L-3**	45994512 (99.64%)	3256170582 (47.35%)	6434822445 (93.58%)	39215758 (85.82%)	1494544 (3.27%)	40710302 (89.09%)
**H-1**	47711662 (99.65%)	3372820782 (47.30%)	6697719051 (93.94%)	41001879 (85.99%)	1722906 (3.61%)	42724785 (89.61%)
**H-2**	36364668 (99.64%)	2556194869 (47.03%)	5078910833 (93.45%)	31131639 (85.77%)	1181501 (3.25%)	32313140 (89.02%)
**H-3**	40330662 (99.68%)	2845385175 (47.21%)	5651182511 (93.77%)	34653151 (85.99%)	1398158 (3.47%)	36051309 (89.46%)

C: 0 μg/L Del; L: 0.5 μg/L Del; H: 5 μg/L Del.

### Identification of DEGs

**Figure 2:**
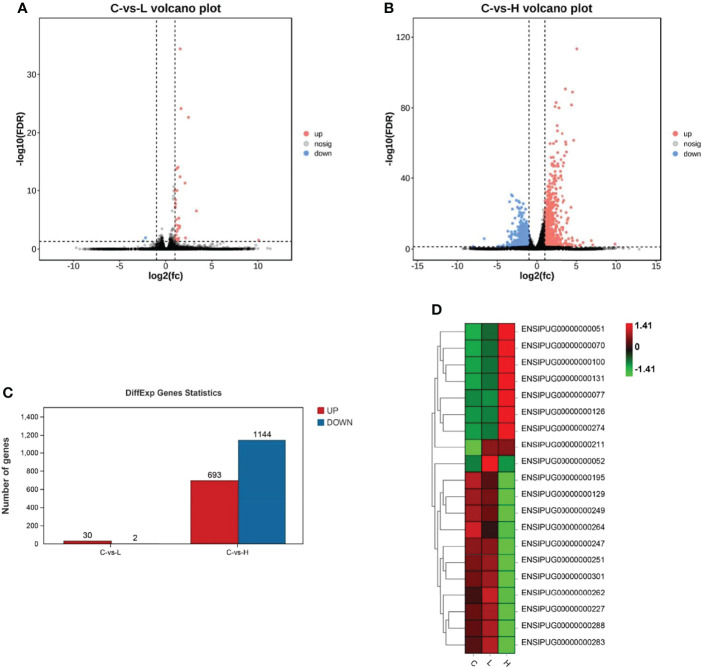
Summary of differentially expressed genes (DEGs) in channel catfish after exposure to 0.5 and 5 μg/L Del. Volcano plots of DEGs in the trunk kidney treated with **(A)** 0.5 and **(B)** 5 μg/L Del. Red and blue dots indicate upregulated and downregulated genes, respectively. **(C)** Statistical results of DEGs in the C (control) vs L (0.5 μg/L), and C (control) vs H (5 μg/L) group comparisons; **(D)** Heat map of gene expression differences.

Based on the differential expression analysis of DESeq2, DEGs between controls (C group) and experimental groups (L and H) were identified and visualized using volcano maps (**Figures 2A, B**). A total of 32 DEGs were detected in the L group and 1837 were detected in the H group (**Figure 2C**; **Tables S1** **,** **S2**). Among these DEGs, 30 were upregulated and 2 were downregulated in the L group. Meanwhile, 693 DEGs were upregulated and 1144 were downregulated in the H group (**Figure 2C**). The differential gene expression map is shown in **Figure 2D**. Moreover, apoptosis-, immune-, and drug metabolism-related DEGs were also detected in the trunk kidney of channel catfish after exposure to Del (**Tables S3** **–** **S5**), which were mainly concentrated in the H group.

**Figure 3:**
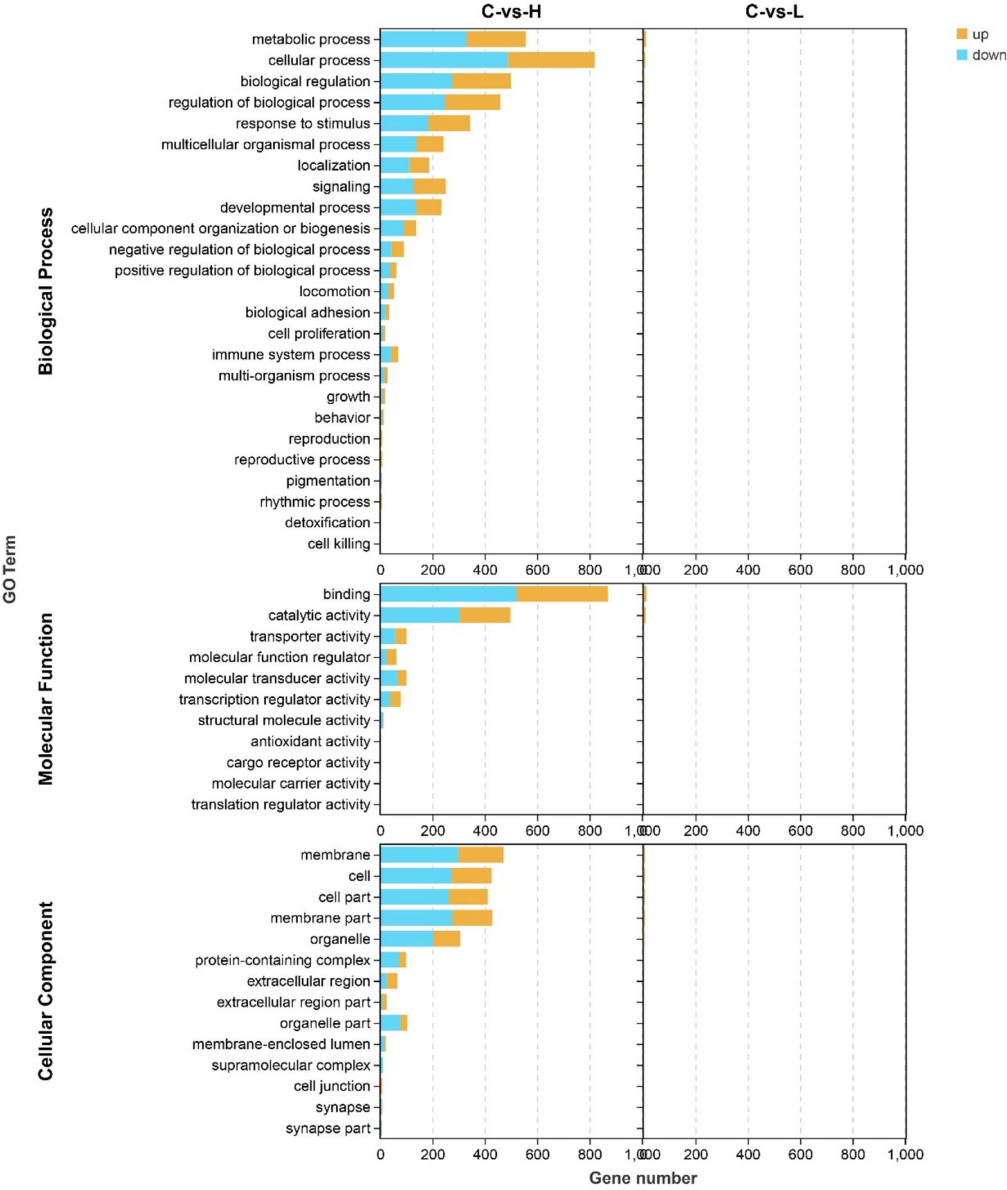
Gene ontology (GO) annotation of differentially expressed genes (DEGs) in the three main GO categories: biological process (BP), cellular component (CC), and molecular function (MF). C: 0 μg/L, L: 0.5 μg/L and H: 5 μg/L.

Next, to better characterize the biological function of DEGs, GO and KEGG pathway enrichment analyses were performed between control and experimental groups. The DEGs in L and H groups were successfully classified into three major groups, including biological processes (BP), cellular components (CC), and molecular functions (MF) (**Figure 3**). The most abundant GO terms in the BP category in the L group were oxidation-reduction process (GO: 0055114), followed by organic hydroxyl compound transport (GO: 0015850) and cholesteric metallic process (GO: 0008203); in the CC category, only four GO terms were detected: plasma lipoprotein particle (GO: 0034358), lipoprotein particle (GO: 1990777), protein-lipid complex (GO: 0032994), and high-density lipoprotein particle (GO: 0034364). In the MF category, the most abundant GO terms were oxidation activity (GO: 0016491) and coenzyme binding (GO: 0050662) (**Table S6**). Only one CC-related term was significantly enriched in the H group, but many terms were significantly enriched in both BP and MF. The GO term significantly enriched in the CC category was MCM complex (GO: 0042555), and the most abundant GO terms in the BP category were negative regulation of biological process (GO: 0048519), followed by negative regulation of cellular process (GO: 0048523), response to organic substances (GO: 0010033), cellular response to organic substances (GO: 0071310) and DNA metallic process (GO: 0006259). In the MF category, the most abundant GO terms were DNA binding (GO: 0003677) and DNA binding transcription factor activity (GO: 0003700) (**Table S6**).

**Figure 4:**
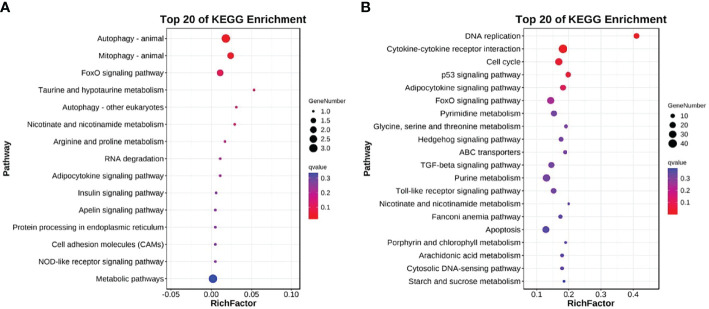
Top 20 KEGG enriched DEGs after exposure to **(A)** 0.5 and **(B)** 5 μg/L Del compared to the control group (0 μg/L Del). The vertical and horizontal axes represent different pathways and rich factors, respectively.

The DEGs were also mapped to the KEGG database to analyze their biological functions and important pathways based on the whole transcriptome background. The KEGG pathway database is mainly divided into five categories: metabolism, genetic information processing, environmental information processing, cell process, and biological system. The KEGG pathway enrichment analysis showed that 15 and 175 pathways were enriched in L and H groups, respectively (**Table S7** **,** **8**). The most significantly enriched pathway in the L group L was Autophagy-animal (KO04140, 3 DEGs) (**Figure 4A**), while in the H group was DNA replication (KO03030, 16 DEGs) (**Figure 4B**).

### Verification With qPCR

**Figure 5:**
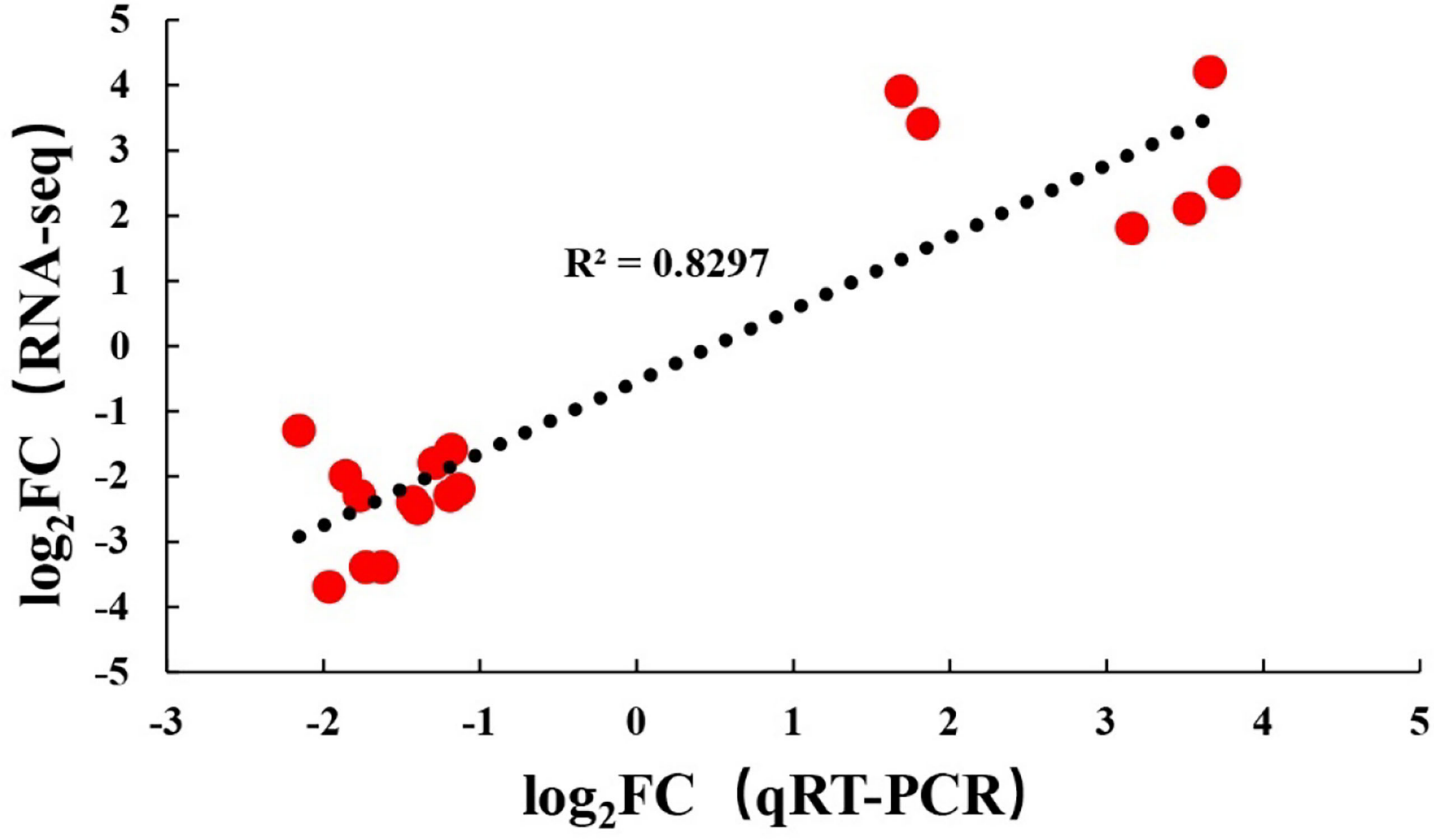
Comparison of levels for 18 DEGs using RNA-Seq and qRT-PCR.

To verify the RNA-seq results, the same RNA samples used for sequencing database construction were used to detect the expression of 18 DEGs by qRT-PCR. The primer sequences of all detected DEGs are shown in **Table 1**. The qRT-PCR results of the 18 DEGs were consistent with the RNA-seq results. These results indicated that the results of RNA-seq were generally reliable (**Figure 5**).

### Sequencing Analysis and Taxonomic Annotation

**Figure 6:**
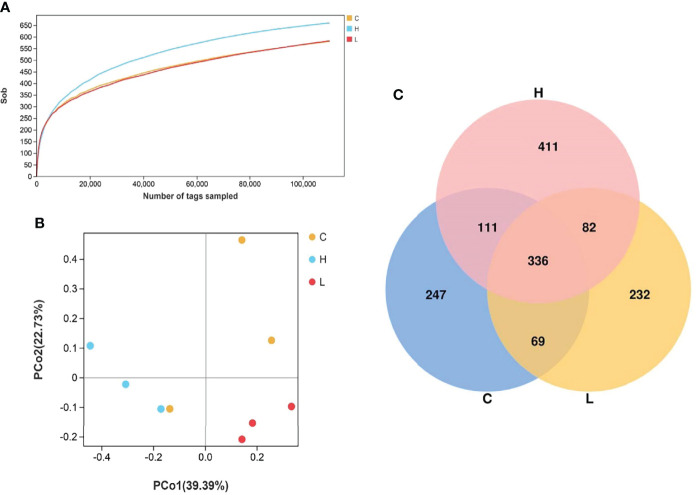
Rarefaction curves of channel catfish intestinal microbial samples **(A)**; Principal component analysis of samples from two groups **(B)**, n = 3; Venn diagram of the OTU distribution **(C)**. C: 0 μg/L Del; L: 0.5 μg/L Del; H: 5 μg/L Del.

A total of 1114159 effective tags were obtained from all intestinal samples of channel catfish. The effective ratio was 92.93-97.38% and the average sequence length was 303 to 478 (**Tables S9** **,** **10**). The sequence sparse curve shows that the sample was sufficient to reflect the community richness and could be used for further data analysis (**Figure 6A**). The PCoA showed that the consistency of the sample was relatively good (**Figure 6B**). The α diversity index of intestinal microbiota after exposure to different concentrations of Del is shown in **Table S11** and did not differ (*p* > 0.05). Additionally, a total of 1488 OTUs were detected using the 97% sequence similarity threshold. There were 336 common OTUs between C, L, and H groups. On the other hand, compared with the C group, 314 unique OTUs were detected in the L group and 493 in the H group (**Figure 6C**).

### Intestinal Microbial Composition

**Figure 7:**
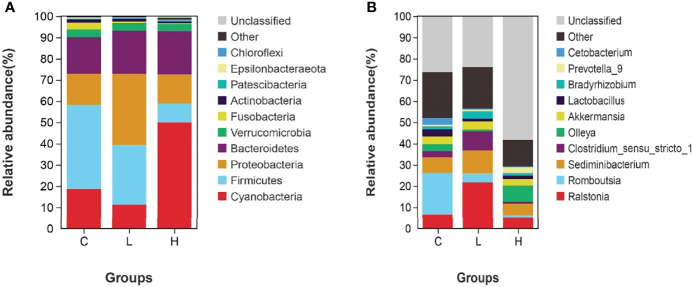
Relative abundances of dominant bacterial phyla **(A)** and genera **(B)** in the intestine of channel catfish after exposure to different concentrations of Del. C: 0 μg/L Del; L: 0.5 μg/L Del; H: 5 μg/L Del.

**Figure 8:**
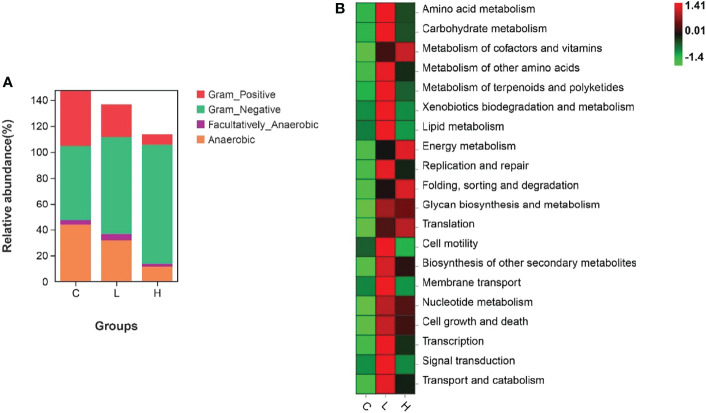
Organism level microbiome phenotypes were predicted by Bugbase **(A)**. Heat map of functional prediction by PICRUSt **(B)**.

Among all intestinal samples, Firmicutes was the main phylum in the top 10, followed by Cyanobacteria, Bacteroidetes, Proteobacteria, Verrucomicrobia, and Fusobacteria (**Figure 7A**). Compared with controls, the abundance of Firmicutes, Cyanobacteria, and Fusobacteria decreased in the L group, while the abundance of Bacteroides, Proteobacteria, and Verrucomicrobia increased. In the H group, the abundances of Firmicutes and Fusobacteria seriously decreased, the abundances of Proteobacteria and Verrucomicrobia slightly decreased, while the abundances of Cyanobacteria and Bacteroidetes significantly increased (**Table S10**). Moreover, the abundances of Firmicutes, Fusobacteria, Actinobacteria, and Epsilonbacteraeota decreased with increasing Del concentrations. At the genus level, the dominant bacteria in the gut of channel catfish included 10 species, such as *Romboutsia*, *Sediminibacterium*, *Ralstonia*, *Akkermansia*, and *Lactobacillus* (**Figure 7B**). After Del exposure, the abundances of *Romboutsia*, *Lactobacillus*, and *Cetobacterium* decreased (**Table S11**). The functional prediction of the bacterial structure showed that the intestinal tract of channel catfish in L and H groups increased the relative abundance of Gram-negative bacteria and decreased the relative abundance of Gram-positive bacteria (**Figure 8A**). Additionally, Del treatment reduced the abundance of anaerobic bacteria in L and H groups. The abundance of aerobic bacteria increased in the L group, while decreased in the H group. The phenotype prediction of the microbiome indicated that these alterations in the bacterial diversity might be involved in different metabolisms as the dominant function (**Figure 8B**).

## Discussion

Large-scale diseases can occur from time to time, resulting in significant economic losses in channel catfish culture (39, 40). Del is not only an important pollutant in water but also an insecticide used in aquaculture, becoming an important factor affecting their healthy cultivation. Therefore, clarifying the toxic mechanisms of Del in channel catfish can provide a theoretical basis for guiding their cultivation and avoiding water pollution. Hence, our current research has important theoretical and practical significance. In the present study, exposure to different concentrations of Del for 6 h led to pathological damage to the intestinal tract and trunk kidney of channel catfish. In the H group, the intestinal villous epithelial cells were disordered and shed, and inflammatory cell infiltration and glomerular atrophy and degeneration were the most serious characteristics, consistent with previous studies (57). The pathological results not only confirmed the toxicity of Del with high concentration to channel catfish but also demonstrated that the immersion experiment was successful, which laid a foundation for subsequent transcriptome and intestinal microbial composition analyses.

Then, we sequenced the transcriptome of the trunk kidney of channel catfish after Del exposure for 6 h. The results showed that the early gene expression of channel catfish was significantly altered after exposure to Del at different concentrations. Only 32 DEGs were enriched in the L group, while 1837 DEGs were enriched in the H group. The enriched DEGs could be divided into three categories: apoptosis, immunity, and metabolism. The L group was mainly enriched in metabolism-related DEGs, such as adobe and p4ha1b, and only two DEGs, such as lgi2a, were significantly downregulated. In the H group, 693 DEGs were upregulated and 1144 DEGs were downregulated, covering the three categories (apoptosis, immunity, and metabolism). The apoptosis-related DEGs enriched in the H group included Ctsl, Gadd45b, Gadd45aa, Caspase-3, Bax, Smad1, Smad6, Smad7, Bcl-2, and TNF superfamily receptors. Moreover, the enriched metabolism-related DEGs included cytochrome P450 and the immune-related ones included the toll-like receptor, chemokine family genes, and IL-1β. These results showed that the low concentration of Del (0.5μg/L) had little effect on channel catfish, but 5μg/L induced a great change in the gene transcription level of the trunk kidney. Hence, the exposure to a high concentration of Del had a strong stimulation on channel catfish in the early stage.

Apoptosis or programmed cell death is considered a key component of homeostasis maintenance and can be induced by various environmental stressors in aquatic organisms, including chemicals (58). Here, we found that channel catfish after 5μg/L Del exposure presented enriched apoptosis-related GO terms, such as tumor necrosis factor receptor binding, tumor necrosis factor receptor superfamily binding, and enriched apoptosis and p53 signaling pathways. Meanwhile, after 0.5μg/L Del exposure the related GO terms and signaling pathways were rarely enriched. Previous studies have shown that Del can induce several types of apoptosis by upregulating the expression of p53 and Bax and inhibiting the expression of Bcl-2 (59, 60). In the current study, p53 and Bax were significantly upregulated, and Bcl-2 was significantly downregulated in the trunk kidney of channel catfish in the H group. Additionally, Del exposure can induce Caspase-3-dependent apoptosis in carp (28). Caspase-3 was also significantly upregulated in the H group. Some studies have also pointed out that cathepsin L (Ctsl) is an important participant in various apoptosis-related pathways by promoting the expression of cytochrome c and bid, and inhibiting the expression of the anti-apoptotic factor Bcl-2 from activating the apoptosis pathway (61, 62). We also found that the Ctsl gene was significantly upregulated but failed to enrich cytochrome c. Moreover, the expression difference of bid was not significant, which might be the problem with the depth of transcriptome sequencing (63, 64). The TGF-β signaling pathway was also enriched in the present study, and the expressions of Smad1, Smad6, and Smad7 were significantly upregulated, which might indicate channel catfish renal fibrosis (65). These results suggested that in the early stage of acute exposure to high concentrations of Del, apoptosis occurred in the trunk kidney of channel catfish, and fibrosis might occur further.

The metabolism comprehends a series of orderly chemical reactions that occur in organisms to maintain life. These reactions enable organisms to grow and reproduce, maintain their structure and respond to the environment. In our research, many metabolism-related DEGs were enriched in channel catfish after Del exposure. Cytochrome P450 (CYP) is involved in the metabolism of exogenous substances (66), especially chemical drugs, and is considered to be a good biomarker to evaluate pollutants in water environments (67). Previous studies have shown that Del exposure can lead to the upregulation of CYP1A1 expression in tuna and rainbow trout (68, 69), and it is time-dependent in rainbow trout (69). Similarly, in the present study, after exposure to 5 μg/L Del, CYP1A1 was also significantly upregulated in channel catfish. CYP1A1 was mainly involved in metabolic pathways and metabolism of xenobiology by cytochrome P450, indicating that CYP1A1 was involved in the metabolism of Del in the trunk kidney of channel catfish. To resist external stimuli, such as pathogenic microorganisms and pollutants, and maintain tissue homeostasis, organisms need to invest a lot of energy, which can disorder energy metabolism (70–72). In the present study, some pathways related to energy metabolism were enriched in channel catfish at the early stage of Del exposure, including lipid metabolism, carbohydrate metabolism, amino acid metabolism, startch and sucrose metabolism, glycolysis/gluconeogenesis, pentose and gluconate interconversion and fructose and mannose metabolism. The results showed that channel catfish provided the required energy through carbohydrate, fatty acid, and amino acid metabolisms to resist the requirements to compensate other functional damage caused by Del toxicity. It also explains that the growth rate of fish is very slow under long-term stress such as pollutants, consistent with previous studies (47, 73). We also found that the expression of myosin, which regulates muscle strength (74), was significantly downregulated, that is, the muscle strength and exercise of channel catfish decreased, consistent with their clinical symptoms. However, we also found that the expression of glucose-6-phosphatase (G6P) was significantly downregulated. G6P can dephosphorylate glucose to promote circulation (47), which means that the energy metabolism of channel catfish was disordered in the early stage of Del acute exposure. However, Hsp70, as a stress protein involved in cell protection and repair (75), was significantly up-regulated. At the same time, it is also enriched in autophagy - animal and mitophagy - animal signal pathways (76, 77), indicating that channel catfish produces an acute stress response under high concentration Del exposure, so as to strengthen cell repair and protection, maintain body homeostasis and improve survival ability.

The fish immune system is very important to the host’s defense, comprehending an important barrier for the body to resist external stimuli such as pathogenic microbial infections (78). Meanwhile, it is also affected by various environmental pollutants, including drugs (79, 80). We showed that in the early stage of acute exposure to a high concentration of Del, the transcriptome of channel catfish trunk kidney was enriched for many immune-related DEGs, including toll-like receptor, chemokine family, and IL-1β. It was also enriched in many immune-related signaling pathways, such as the Toll-like receptor, RIG-I-like receptor, and C-type lectin receptor signaling pathways. The TLR family senses the molecular signatures of microbial pathogens and plays a fundamental role in innate immune responses (81). The expressions of TLR9, TLR21, TLR18, TLR1, TLR5, and TLR19 in channel catfish were significantly downregulated in the early stage of Del acute exposure, which led to the decline of the body’s ability to deal with pathogenic microorganisms. Additionally, alkaline phosphatase (ALP), chemokines, nucleotide-binding oligomeric domain (NOD), and integrin play an important role in innate immunity by promoting inflammation and antibacterial responses and recruiting leukocyte phagocytosis (48, 82–86). However, we found that the expressions of alkaline phosphatase (ALP), CCL34, CXCR5, NOD1, NLRC3 and integrin alpha in were significantly downregulated channel catfish after Del exposure. Along with the enriched immune-related signaling pathways, these results indicated that the immune system was seriously affected by Del exposure. Further, we showed that Del could inhibit the immune defense system of channel catfish, especially the innate immunity. Similar results were detected for snakehead, Chinese rare minnow, and goldfish (12, 24, 31, 87). Therefore, Del can reduce fish immunity and make it more vulnerable to pathogen infections, consistent with the result that copper sulfate stimulation can make channel catfish more vulnerable to *Edwardsiella ictalurid* (88).

Surprisingly, in the early stage of high concentration Del exposure, C3, iNOS, and IL-1β were significantly upregulated. IL-1β is a powerful proinflammatory factor (89) and iNOS is an important index to measure inflammation (28, 90). Their upregulation expression can represent the body’s inflammation to protect itself in response to Del toxicity. However, the pathological results showed that there was no clear inflammation in the trunk kidney, which might be related to the fact that fish were still in the early stage of exposure and the inflammation did not result in pathological changes yet. Additionally, the complement system can mediate host immune and inflammatory responses and play a key role in preventing pathogen infections (91), indicating that under Del toxicity, the complement system can enhance the innate immunity of channel catfish through compensation, similar to gilthead seabream (*Sparus aurata*) (68). However, after subacute exposure to Del, the levels of C3, C4, and other immune parameters significantly decreased in snakeheads (24). A potential explanation for this difference might be that the effects of Del on aquatic animals are species and dose-dependent (92), that is, Del can present considerable toxicity differences among different species (24, 93, 94). Overall, in the present study, the conflicting results regarding the transcription level of immune-related genes are likely to reflect the complexity of the immune defense response of channel catfish after acute exposure to high concentrations of Del.

Under normal circumstances, beneficial and harmful bacteria are in a dynamic balance in the intestine (95). Here, in the early stage of Del exposure, significant changes were detected in the composition of the intestinal microbiota of channel catfish, with a significant decrease in Gram-positive bacteria and a significant increase in Gram-negative bacteria. The intestinal microbiome composition analysis of channel catfish showed that the dominant bacteria at the phylum level were Firmicutes, Cyanobacteria, Bacteroides, Proteobacteria, Verrucomicrobia, Fusobacteria, and Actinobacteria. Previous studies have also reported similar results, showing that the intestinal flora of fish is mostly composed of Proteobacteria, Firmicutes, Fusobacteria, and Actinobacteria (96, 97).

Furthermore, Del exposure significantly reduced the abundance of Firmicutes, Fusobacteria, and Actinobacteria, and the Firmicutes/Bacteroidetes ratio also decreased. Bacteroides and Firmicutes play an important role in energy metabolism in the fish intestine. Bacteroides can encode carbohydrate-related enzymes involved in the hydrolysis of glycoconjugates. Firmicutes promote the intestinal absorption and metabolism of fatty acids and play an active role in the growth performance, immunity, digestion, and disease resistance of aquatic animals (98–101). The microbiome phenotype prediction showed that changing bacterial species might participate in many metabolisms as the dominant function. The changes in the number of Bacteroides and Firmicutes caused by Del exposure might also lead to energy metabolism disorders in channel catfish. The transcriptome data showed that channel catfish provided the required energy to resist Del toxicity through carbohydrate, fatty acid, and amino acid metabolisms. The changes in intestinal microbial composition showed that they mainly rely on nutrients *in vivo* to make up for the massive consumption of energy, rather than exogenous intake. We also found that the abundance of Fusobacteria and Actinobacteria significantly decreased, which can increase the risk of infections (102, 103). Hence, Del exposure in the early stage can make the body more vulnerable to pathogen infections and cause metabolic disorders.

At the genus level, there were 10 dominant bacteria, including *Romboutsia*, *Sediminibacterium*, *Ralstonia, Akkermansia, Lactobacillus*, *Olleya*, and *Cetobacterium*. Among them, the abundance of *Romboutsia*, *Lactobacillus*, and *Cetobacterium* significantly decreased with increasing Del concentrations. However, *Lactobacillus* and *Cetobacterium* are important beneficial bacteria in the intestine (104). For example, *Lactobacillus* can reduce the intestinal pH, which might contribute to overcoming pathogen challenges (105). *Cetobacterium* might be involved in the metabolism of the host and provide vitamin B12 (106). Therefore, the intestinal function of channel catfish might also be affected in the early stage of Del exposure. These results suggested that Del might adversely affect the metabolism and immune function of channel catfish by changing the intestinal microbial community in the early stage of acute exposure.

In summary, the pathological results showed that a high concentration of Del can damage the intestine and trunk kidney of channel catfish in the early stage. Through transcriptome analysis, we detected several DEGs related to immunity, metabolism, and apoptosis in the trunk kidney of channel catfish, indicating that Del exposure in the early stage can also lead to immunotoxicity, metabolic disorders, and tissue damage. Additionally, the 16S rRNA gene sequencing showed that exposure to Del can significantly change the composition of the intestinal microbial community, interfering with the normal metabolism and immune function of the intestine of channel catfish. Overall, we analyzed the adverse effects of Del on the early exposure of channel catfish and showed why it can trigger infectious diseases and slow growth in aquaculture.

## Data Availability Statement

The datasets presented in this study can be found in online repositories. The names of the repository/repositories and accession number(s) can be found below: https://www.ncbi.nlm.nih.gov/genbank/, PRJNA820141 https://www.ncbi.nlm.nih.gov/genbank/, PRJNA820136.

## Ethics Statement

The animal study was reviewed and approved by the Animal Welfare and Research Ethics Committee of Yangtze River Fisheries Research Institute (YFI2021YYB002). Written informed consent was obtained from the owners for the participation of their animals in this study.

## Author Contributions

YY and XA conceived and designed the study. YY and XZ performed most of the experiments. XA, XZ, YH, HZ, NX, YL, and GF provided bioinformatics assistance and support. HZ and YY wrote the manuscript. All authors proofed approved the manuscript.

## Funding

This work was supported by Central Public-Interest Scientific Institution Basal Research Fund CAFS(2020TD11), Central Public-interest Scientific Institution Basal Research Fund (NO.YFI202209) and Yancheng fishery high quality development project (No.YCSCYJ2021026).

## Conflict of Interest

The authors declare that the research was conducted in the absence of any commercial or financial relationships that could be construed as a potential conflict of interest.

## Publisher’s Note

All claims expressed in this article are solely those of the authors and do not necessarily represent those of their affiliated organizations, or those of the publisher, the editors and the reviewers. Any product that may be evaluated in this article, or claim that may be made by its manufacturer, is not guaranteed or endorsed by the publisher.
